# A comparative assessment of *RNF38* and *P53* genes expression in the sperm samples obtained from males with normozoospermia and asthenospermia: A case-control study

**DOI:** 10.18502/ijrm.v20i12.12563

**Published:** 2023-01-09

**Authors:** Alireza Alizadeh, Sina Mirzaahmadi, Golnaz Asaadi Tehrani, Neda Jabbara

**Affiliations:** Department of Genetics, Faculty of Basic Sciences, Islamic Azad University, Zanjan Branch, Zanjan, Iran.

**Keywords:** RNF38, P53, Asthenozoospermia, Motility.

## Abstract

**Background:**

Infertility is considered as a common problem appears in about 10-12% of couples in their reproductive ages*. *Ring finger protein 38* (RNF38)* gene is a ubiquitin-protein ligase that can regulate Protein 53* (P53) *and affect cellular motility.

**Objective:**

Considering the role of *P53* on cellular motility and *RNF38* on the regulation of *P53*, the present study aimed to assess the difference between *RNF38* and *P53* genes expression in normozoospermic and asthenospermic samples as a diagnostic biomarker in males.

**Materials and Methods:**

The present study was conducted among 21 asthenospermicsand 63 healthy individuals. First, the real-time polymerase chain reaction technique was applied to measure the expression level of the *P53* and *RNF38* genes extracted from sperm samples, and the glyceraldehyde-3phosphate dehydrogenase gene was selected as the reference gene.

**Results:**

An increase and a decrease occurred in the level of *P53* and *RNF38* genes expressions in asthenospermic and normozoospermic samples, respectively. In addition, a significant difference was observed between increasing *P53 *gene expression (p < 0.001), reducing *RNF38* one, and decreasing sperm motility (p < 0.001) in asthenospermic cells compared to that of normozoospermic ones.

**Conclusion:**

Based on the results, an increase in the expression of the *P53* gene and a decrease in the expression of the *RNF38* gene had a significant relationship with asthenospermia in men. Therefore, it is expected that an effective step should be adopted to diagnose the asthenospermia expression pattern by using these results.

## 1. Introduction

Based on the world health organization report, about 10-12% of couples worldwide are suffering from infertility, half of which are related to males. Although there is no clear reason for male infertility in 30-40% of cases, the other 50% are related to sperm-related disorders (1-3) including asthenozoospermia or decreasing sperm motility in males medically (4) which appears because of disturbing genital system development and decreasing sperm motility and fertilization capacity by genetic disorders (5). Sperm-specific RNAs are important biomarkers in the steps related to germ cells and the share of somatic cells. Thus, these RNAs can be considered valuable diagnostic markers for fertility, sperm viability, and motility, and can be used as a predictor for prognosis during in vitro fertilization (6-8).

The human Protein 53 (*P53*) gene is located in the short arm of chromosome 17 (17p139) (9) and encodes the *P53* protein, which contains 393 amino acids (10, 11). Numerous studies represented the importance of the function of *P53* in apoptosis in preventing tumor growth. The *P53* gene plays a role in many cellular processes such as metabolism, antioxidant response, and DNA repair (12, 13).

Ring finger protein 38 (RNF38) is located in the chromosome (9p13) of a human. Additionally, as a ubiquitin-protein ligase, it can ubiquitinate and relocate *TP53*/*P53* to promyelocytic leukemia protein nuclear body-associated foci and plays an important role in regulating *P53*. The *RNF38* gene has widely expressed in different types of human tissues. Based on recent observations, *RNF38* is considered one of the *P53*-connected proteins (14, 15). E3 disturbs the function of tumor suppressor genes or oncogene, which participates in cellular transduction and tumor progression (16, 17). The main role of *P53* is to provide signals since it can direct cells toward repairing or stopping growth, or even apoptosis by inducing the expression of a series of genes. *P53* is a central factor in cells, and RNF38 is a P53-regulatory factor with inhibitory activity on RNF38.

Since no study has been done regarding the effect of *P53* and *RNF38* genes in asthenozoospermia, our purpose was whether *P53* is involved in asthenozoospermia is an important process and whether its involvement is affected by *RNF38*. We sought to find out whether *P53* affects motility through *RNF38* or not. Thus, the expression of 2 genes was evaluated in the present study.

Additionally, the present study aimed to assay the level of DNA fragmentation by using a Sperm DNA fragmentation assay (SDFA) kit. Assessing and recognizing this relationship can improve the efficiency of therapy, identifying new molecular biomarkers for the early diagnosis of asthenospermia in males, evaluating optimal therapeutic approaches, and diagnosing asthenozoospermic samples by assaying sperm in males.

## 2. Materials and Methods

In this case-control study, the count and motility of the sperm samples of the 84 participants, who were referred to Bu Ali Laboratory in Zanjan, Iran for 6 months from June-November 2020, were written on the day of sample preparation and the data were sorted from minimum motility to maximum one.

Out of 84 samples that were assessed in the present study, 21 and 63 were related to the asthenospermics and healthy individuals, respectively. In this regard, 2-3 ml of sperm was prepared from each participant and tested immediately. The HFTCASA computer-aided semen analysis system software (8.00) was used to analyze the motility rate of sperm samples.

Also, the normal group was classified into 3 subgroups (n = 21/each) to conduct the same statistical comparison among the subgroups, and with a 21-member asthenospermia group. In this regard, the sperm motility rate of 3 subgroups A, B, and C were considered, including (A: 41.03-57.14), (B: 58.22-67.82), and (C: 68.55-82.46), respectively.

### RNA extraction

RNA was extracted from collected samples using an RNA column extraction Kit (BioBasic Inc., America) and purified.

### cDNA synthesis

Takara kit (TAKARA BIO INC., Japan) was used to synthesize cDNA from extracted RNA. In the present study, the real-time polymerase chain reaction (PCR) technique was applied to assess the relationship between *P53* and *RNF38* genes expression in the sperm samples related to the males with normospermia and asthenospermia. Table I represents the sequence of the primers used for *P53* and *RNF38* genes.

The intended real-time was conducted in the final volume of 20 μl, including SGq PCR MasterMix 2x with the concentration of 1X (Parstous) and 10 pmol of each primer (Gen fanavaran, Iran), and 0.1 ml of the total volume of cDNA. The program of real-time PCR included one cycle of primary denaturation (94 C; 5 min), 30 cycles of denaturation (94 C; 30 sec), 30 cycles of annealing (52 C; 30 sec), 30 cycles of extension (72 C; 30 sec), and 1 cycle of final amplification (72 C; 5 min). All of the values obtained from tests were normalized by the expression level of the glyceraldehyde-3-phosphate dehydrogenase* (GAPDH)* gene as an internal control. The graph analysis, which represents gene expression, was performed to ensure the conduction of real-time PCR. Additionally, an SDFA kit (Halotech, Spain) was used to assay the level of DNA fragmentation. Sperm DNA fragmentation (SDF) levels were assessed using HalospermⓇ G2 kit (HT-HSG2, Halotech, Spain) as the guideline of the company for SDFA. In this regard, sperm DNAs with halo < 15%, 15-30%, and > 30% possessed very high, fair, and poor fertility potential.

**Table 1:** Primer sequence for GAPDH, *P53*, and *RNF38* genes


**Primer**	**Primer sequence**	**Length of a created piece**
**Forward (GAPDH)**	5'-GGTCATCATCTCTGCCCCCT-3'	
**Reverse (GAPDH)**	5'-AGGCAGGGATGATGTTCTGG-3'	*276BP*
**Forward (RNF38)**	5'- AAGCAGATATTGAACAACTTCCTTC-3'		
**Reverse (RNF38)**	5'- CTTAAGCCATTTGTCAACACACTT-3'	*176BP*
**Forward (P53)**	5'- ATAGTGTGGTGGTGCCCTATGAGC-3'		
**Reverse (P53)**	5'- TTCCAGTGTGATGATGGTGAGGAT-3'	*134BP*
*GAPDH*: Glyceraldehyde-3phosphate dehydrogenase, RNF38: Ring finger protein 38, P53: Protein 53		

### Ethical considerations

The project was found to be under the ethical principles and the national norms furthermore, all the participants completed the consent form, and standards for conducting medical research in Iran and was approved by the Ethics Committee of Islamic Azad University of Zanjan, Zanjan, Iran (Code: IR.IAU.Z.REC.1397.064).

### Statistical analysis 

REST 2009 software, developed by M. Pfaffl (Technical University Munich) and QIAGEN for gene expression analysis (v 2.0.13), was applied for evaluating the results of SDFA. Further, the results were analyzed and compared to motility using Sperm Analyzer System (HFTCASA) computer-aided semen analysis system software, 8.00. Furthermore, the expression of 2 *RNF38* and *P53* genes were comparatively assessed using IBM SPSS Statistics 26, and the significance level of 0.05 was considered statistically significant. A *t* test was implemented to compare the results by IBM SPSS Statistics 26 software, which was evaluated using the Kolmogorov Smirnov test. To compare 2 groups from *t* test and to compare 3 groups from one way analysis of variance.

## 3. Results

Based on the comparative assessment of *P53* and *RNF38* genes expression in asthenozoospermia regarding participants and that of the normal subgroup of A, an increase and decrease were observed in the expression level of *P53* and *RNF38* genes in the patients compared to that of normal individuals, respectively. Furthermore, there was a significant difference between *P53* and *RNF38* gene expression. Accordingly, an increase in the *P53* gene expression of males with asthenozoospermia compared to that of normal individuals could partly affect the reduction of sperm motility.

After comparing *P53* and *RNF38* gene expression in the asthenospermic and the normal subgroup of B, the expression level of *RNF38* and *P53* genes in participants decreased and increased compared to that of normal individuals, respectively. In addition, both groups observed a significant difference regarding *P53* gene expression.

Thus, a decrease in *RNF38* gene expression results in increasing *P53* and reducing sperm motility.

Regarding the comparative assessment of *P53* and *RNF38* genes expression in patients and that of the normal subgroup of C, an increase and decrease were observed in the expression level of *P53* and *RNF38* genes in the males with asthenozoospermia compared to that of normal individuals, respectively. Additionally, *P53* and *RNF38* gene expression in both groups was significantly different from the reduced motility group, respectively. Thus, decreasing the expression of the *RNF38* gene as a ubiquitinating protein can influence sperm motility by increasing *P53* gene expression. The increase in the *P53* expression of males can be used as a molecular biomarker that is sensitive to variations in sperm motility.

*P53* and *RNF38* gene expression were compared by representing that *RNF38* and *P53* gene expression in the asthenospermics decreased and increased significantly compared to normal individuals. Furthermore, a significant difference was observed between the expression level of these genes and their relationship with sperm motility.

The results of SDF analysis with REST (2009) software indicated that the expression of both *RNF38* and *P53* genes in poor cells was higher than in good ones, which indicates a significant increase in the expression of the *RNF38* gene (p = 0.01), while the increased expression was insignificant in *P53* gene (p = 0.81).

As shown in table II, a significant difference was observed between the 2 normal and case groups. Thus, an increase in *P53* gene expression reduced sperm motility and consequently, asthenospermia in males by decreasing the expression and ubiquitination activity of *RNF38*.

**Figure 1:**
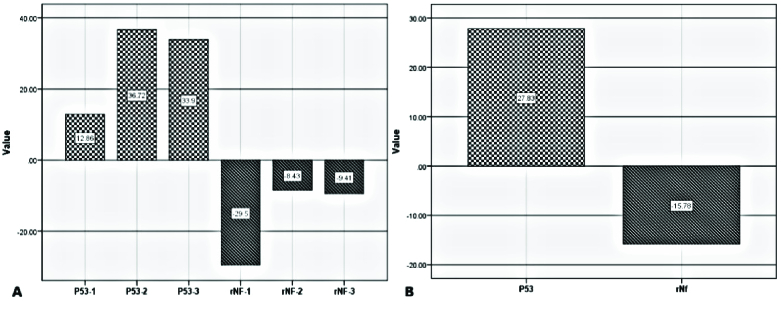
The column chart shows the mean difference of normal and case groups regarding *P53* and *RNF38* genes expression. A) The mean difference of gene expression between each subgroup A, B, and C separately with patients; (subgroup A: P53-1, rNF-1), (subgroup B: P53-2, rNF-2) and (subgroup C: P53-2, rNF-2). B) The mean difference of gene expression between normal samples only with patients.

To identify which of the subgroups have the most mean difference with patients, as shown in figure 1, the mean difference of normal group regarding *P53* and *RNF38* was analyzed separately (the expression of each subgroup of normal samples) and total (the mean expression of normal sample) with patients.

**Figure 2:**
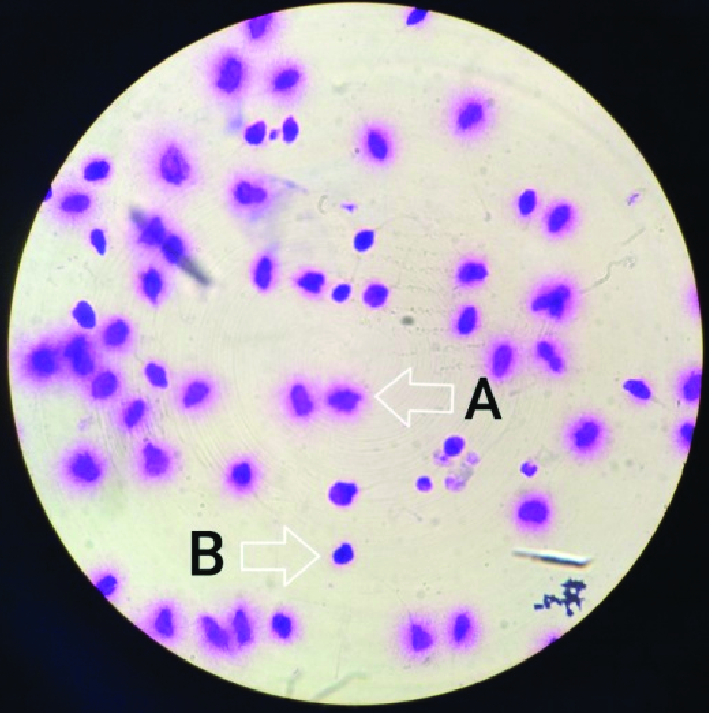
Sperms (A) with halo (healthy and without DNA fragmentation) and (B) without halo (with DNA fragmentation) at × 100 magnification.

As observed in figure 2, 35.70% of the samples possess fragmented DNA without a halo representing poor fertility. Regarding the evaluation of 24 samples, comparing gene expression with the level of SDF, and considering the obtained p-value, asthenospermia is more related to *P53* and *RNF38* gene expression and motility than DNA fragmentation.

### SDFA results

Based on the results in SDF (Table III), an increase in *RNF38* expression in poor cells was more than good, indicating a significant increase in expression (p = 0.01). In addition, an increase in *P53* expression in poor cells was more than good, indicating an increase in expression, although it was insignificant (p = 0.81).

**Table 2:** Mean difference of expression level of *P53* and *RNF38*


**Gene name**		**Mean difference**	**P-value**
***P53***		27.83	0.00
	**Subgroup A**	12.86	0.02
	**Subgroup B**	36.72	0.02
	**Subgroup C**	33.90	0.00
***RNF38***		-15.78	0.00
	**Subgroup A**	-29.50	0.01
	**Subgroup B**	-8.43	0.00
	**Subgroup C**	-9.41	0.03
To compare 2 groups from *t *test and to compare 3 groups from one way analysis of variance. *P53*: Protein 53, *RNF38*: Ring finger protein 38			

**Table 3:** The SDFA results concerning gene expression in 2 groups


**Gene**	**Reaction efficiency**	**Expression**	**P-value**	**Result**
***GAPDH***	0.7219	1.000	
***P53***	0.6367	1.498	0.81	
***RNF38***	0.6376	23.335	0.01	UP
GAPDH: Glyceraldehyde-3phosphate dehydrogenase, P53: Protein 53, RNF38: Ring finger protein 38				

## 4. Discussion

Male infertility is considered the reason for 40-50% of infertilities (18, 19), which is found in about 7% of males. The etiology of infertility in various areas is different by considering diverse geographical, human, sanitary, and cultural conditions. Determining the factors affecting infertility in different countries by considering geographical conditions is important. Testicle-related factors, varicocele, a genetic defect in the Y chromosome, genetic abnormalities, epigenetics, asthenozoospermia, oligospermia, and idiopathy are the most important factors related to male infertility. The *P53* tumor suppressor gene regulates the cell cycle in G1-S and S2-M, inducing apoptosis and responding to severe DNA damage. *RNF38* gene modifies a nuclear ubiquitin protein, leading to a change in *P53*.

Further, it is regarded as an applied ubiquitin-protein ligase and *P53*-connected partner. Thus, it plays an important role in regulating the *P53* gene. Based on the results, the *P53* tumor suppressor gene expression possesses diagnostic and prognostic value in assessing the reason for male asthenozoospermia (13-20).

The present study assessed the expression level of *RNF38* and *P53* genes in the sperm cells collected from males with asthenozoospermia and normozoospermia. Given that motility represents the level of sperm motility and lack of motility or its low level can be regarded as one of the reasons for asthenozoospermia. The *P53* gene plays a role as a tumor suppressor gene, the expression of which increases in most diseases and abnormalities, and *RNF38* gene is involved in different cellular processes such as cell proliferation and cycle, apoptosis, and DNA repair (21), *RNF38* ubiquitins *P53* through ubiquitin ligase activity, leads to a decrease in its expression. Further, the excessive expression of *RNF38* can result in changing *P53* expression. Results from the previous study indicated that *P53* plays an effective role in cell motility, invasion, and metastasis (22-27). A better understanding of the mechanism related to the effects of *P53* on cell motility and invasion leads to approaches for modifying *P53*-related abnormalities (28). Based on the present study results, sperm motility is related to the expression level of the *P53* gene, and an increase in expression results in decreasing sperm motility. In addition, the reduction in motility was significantly related to an increase in *P53* gene expression and a decrease in *RNF38*. Accordingly, an increase in *P53* gene expression and a decrease in *RNF38* may reduce motility, which is significantly related to asthenozoospermia in males.

Based on the comparative assessment of *P53* and *RNF38* genes expression, an increase occurred in the expression level of the *P53* gene related to the sperm cells of individuals with asthenozoospermia compared to that of individuals with normozoospermia in the subgroups of A, B, and C, as well as a total of group D. Further, a significant difference was observed between the expression and decreasing *RNF38* gene and motility, and consequently asthenozoospermia in males (p < 0.001). Further, the *RNF38* gene expression decreased in the intended comparison by representing a significant difference regarding its expression level between the males with asthenozoospermia and that of normal individuals in subgroups A and B. Furthermore, a significant difference was reported between the expression of this gene in the males with asthenozoospermia and that of the males with normozoospermia, its relationship with increasing *P53* gene expression, and consequently a decrease in sperm motility (p < 0.001).

A similar study was conducted on the effect of oxidative stress and the level of P53 protein in the sperm of males with asthenozoospermia and normozoospermia, and found that its level in the asthenozoospermia group is significantly more compared to that of the normal group (p < 0.001) and the level of motility is significantly related to *P53* gene expression (p < 0.001) (15), which are consistent with the results of the present study.

*RNF38* can modify *P53* in conditions such as in vivo and in vitro. Although numerous studies were conducted by different researchers on *P53* and *RNF38* genes expression in the cancer field, few studies were performed regarding the relationship between the expression of these genes and sperm motility and infertility.

On focusing on the expression of the *RNF38* gene and the development of lung cancer, it was found that increasing the expression of *RNF38* is significantly associated with metastasis to the lymph nodes in lung cancer, which can be considered as evidence of the effect of *RNF38* expression in cellular motility (29).

The rate of *RNF38* expression increased in liver carcinoma cells. An increase in *RNF38* expression is related to cancer invasion and progression, as well as the inhibition of apoptosis in vivo and within the body (29, 30). In addition, in line with the present study results, we examined *P53* and its mechanism in prostate cancer and observed that prostate cancer cells have low *P53* expression (p > 0.05), leading to an increase in the migration and adhesion of PC cells by inhibiting JNK and ERK activation. Another study evaluated the relationship between *P53* and Mmp-2 and found a significant association with the prognosis of metastatic lung cancer spinal cord tumors. Thus, *p53* plays a role in cellular motility and increases cell migration by increasing RHO (Rho family of GTPases) pathway activity, indicating the effective role of *p53* in sperm cell motility (31).

## 5. Conclusion

The present study results represented a significant relationship between *P53* and *RNF38* gene expression and asthenospermia among males and changing their expression level in asthenospermic cells. Further, an increase in P53 expression in asthenospermic cells is significantly associated with decreased sperm motility. The present study could provide further research on the relationship between *P53* and *RNF38* gene expression alteration with motility in asthenospermia in males at a scale larger than both case and control groups.

##  Conflict of Interest

The authors declare that they have no known competing financial interest or personal relationships that could have influenced the work reported in this manuscript.
